# 

**Published:** 1873-04

**Authors:** 


### TABLE OF CONTENTS.

ORIGINAL COMMUNICATIONS.

Yellow Fever. By T. A. Cook, M.D., . . . . . . . . . . 193
The Prevalent Fever of Cherokee-Georgia. By Robert Battey, M.D., . 201
Medical Extracts: Practical and Philosophical—No. 1. By L. B. Anderson, M.D., . . . . . . . . . . . . . . . . . . 205
Correspondence. By Wm. R. Whitehead, M.D., . . . . . . 208

SELECTIONS.

Purulent and Gonorrhœal Conjunctivitis. By Professor Seely, . . . 212
A New Treatment for Cystitis and Irritable Bladder of Females. By M. Yarnall, M.D., . . . . . . . . . . . . . 216
Philadelphia County Medical Society, . . . . . . . . . . 218
Eucalyptus Globulus in Intermittent Fever, . . . . . . . 221
Treatment of Gonorrhœa, . . . . . . . . . . . . 223
Sulphites in Tonsilitis, . . . . . . . . . . . 224

EXTRACTS AND GLEANINGS.

An Old Remedy in a New Rolé—A Substitute for Quinine—Vascular Cornea-Pannus, Granulated Lids, 225; Hooping-Cough, 226; Arsenic in Cutaneous Diseases; On the Internal Use of Carbolic Acid, 228; Treatment of Gonorrhœa, 229; Black Cohosh, 230; The Science and Art of Healing Wounds, 231; Colotomy for Intestinal Obstruction, 232; Treatment of some Affections of the Nasal Cavities—Hemorrhage from the Nose, 233; Conservative Surgery in Minor Operations—Nutritive Enemata, 234; Antiseptic Injections and Drainage Tubes in Empyema—Gendret's Blistering Ointment, 235; On Vaccination and Revaccination of Pregnant Women—Cimicifuga in Enlargement of Spleen, 236; Sulphurous Acid in the Treatment of Contused Wounds—Treatment of Irritability of the Stomach, 237; The Fever Tree—Carbazotate of Ammonia in the Treatment of Malarial Poisoning—Treatment of Hemorrhage from the Bowels in Typhoid Fever, 238; On Thoracentesis—Lobelia in Small Doses—Ichthyosis Cured by Sulphate of Copper, 239; Strumous Ophthalmia in Children—Syrupus Cubebæ—Treatment of Erysipelas by the Application of Camphor in Ether, 240; Change of Life in Health and Disease—The Conjoint Use of the Sulphates of Cinchona and Quinine, 241; Cauterization of the Urethra—Alterative Syrup—Orchitis Treated with Absolute Rest, 242; Bromides in the Summer Complaints of Children—Delirium Tremens—Impotence, 243; Contagiousness of Purulent Secretions—Nasal Hemorrhage—Nitrate of Silver in Jaundice and Chronic Gastritis, 244; Chorea—Chloral and Strychnia—The Properties of Horse-Mint, 245; Treatment of Diarrhœa in Infants—Aperients in Eczema—Epilepsy Cured by the Use of Bromide of Potassium and Sulphate of Atropia—Treatment of Tic-Douloureux by Ice, 246; On the Employment of Nux Vomica and the Salts of Strychnine in the Treatment of Obstinate Vomiting—Acute Sclerotitis and Iritis—Catarrh Cut Short—Nervous Sick Headache—Removal of Stains of Nitrate of Silver, 247; How to Take a Cold Bath—Expectorant Mixture—Pulmonary Consumption—Poisoning by Nitrate of Silver—For Itching of the Anus—Locomotor Ataxia, 248; Acute Traumatic Phlebitis—Hydrate of Chloral in Pertussis—Management of Scabies—Carbolic Acid as a Local Anæsthetic—Dressing for Stumps—Diarrhœa, 249.

EDITORIAL AND MISCELLANEOUS.

Georgia Medical Association—Fulton County Medical Society—American Medical Association, . . . . . . . . . . . . . . 250
Dr. S. S. Satchwell—Kind Words and Suggestions, . . . . . . 251
Lowndes County (Mississippi) Medical Society—Charleston Medical Journal and Review, . . . . . . . . . . . . . . . . 252
Communications—Cincho-Quinine—The Popular Science Monthly, . . . 253
Medical Register and Directory of the United States, . . . . . . 254
List of Payments to the Record, Commencing with the January Number, . 255

